# Differential Response of Two Human Breast Cancer Cell Lines to the Phenolic Extract from Flaxseed Oil †

**DOI:** 10.3390/molecules21030319

**Published:** 2016-03-08

**Authors:** Angela Sorice, Eliana Guerriero, Maria Grazia Volpe, Francesca Capone, Francesco La Cara, Gennaro Ciliberto, Giovanni Colonna, Susan Costantini

**Affiliations:** 1 CROM, Istituto Nazionale Tumori “Fondazione G. Pascale”-IRCCS, Napoli 80131, Italy; a.sorice@istitutotumori.na.it (A.S.); e.guerriero@istitutotumori.na.it (E.G.); f.capone@istitutotumori.na.it (F.C.); 2 Istituto di Scienze dell'Alimentazione, CNR, Avellino 83100, Italy; mgvolpe@isa.cnr.it; 3 Istituto di Biologia Agro-ambientale e Forestale, CNR, UOS di Napoli, Napoli 80131, Italy; francesco.lacara@cnr.it; 4 Direttore Scientifico, Istituto Nazionale Tumori “Fondazione G. Pascale”-IRCCS, Napoli 80131, Italy; g.ciliberto@istitutotumori.na.it; 5 Servizio di Informatica Medica, Azienda Ospedaliera Universitaria, Seconda Università di Napoli, Napoli 80138, Italy

**Keywords:** flaxseed, phenolic extract, breast cancer cell lines, gene regulation

## Abstract

Many studies have evidenced that the phenolic components from flaxseed (FS) oil have potential health benefits. The effect of the phenolic extract from FS oil has been evaluated on two human breast cancer cell lines, MCF7 and MDA-MB231, and on the human non-cancerous breast cell line, MCF10A, by SRB assay, cellular death, cell cycle, cell signaling, lipid peroxidation and expression of some key genes. We have evidenced that the extract shows anti-proliferative activity on MCF7 cells by inducing cellular apoptosis, increase of the percentage of cells in G0/G1 phase and of lipid peroxidation, activation of the H2AX signaling pathway, and upregulation of a six gene signature. On the other hand, on the MDA-MB2131 cells we verified only an anti-proliferative activity, a weak lipid peroxidation, the activation of the PI3K signaling pathway and an up-regulation of four genes. Overall these data suggest that the extract has both cytotoxic and pro-oxidant effects only on MCF7 cells, and can act as a metabolic probe, inducing differences in the gene expression. For this purpose, we have performed an interactomic analysis, highlighting the existing associations. From this approach, we show that the phenotypic difference between the two cell lines can be explained through their differential response to the phenolic extract.

## 1. Introduction

Recent independent findings have assessed that dietary phenols have been shown to possess anti-cancer properties by acting as key modulators of signaling pathways and, therefore, they can be considered as chemo-preventive agents [1,2]. This is supported by epidemiological studies, which suggest that a high consumption of plant derived foods decreases the incidence rates of various cancers [1,2]. However, the cellular mechanisms by which the phenols elicit these anti-cancer effects are multifaceted because they include the regulation of growth factor-receptor interaction and the cell signaling cascades such as the altering of the redox state of the cell, which modulate the expression of genes involved in cell cycle arrest, cell survival and apoptosis through kinases and transcription factors [1]. Recently, the phenols resulted to contribute to epigenetic changes by inducing post-translational modifications and changes of microRNA expressions [2].

For centuries, flax (*Linum usitatissimum*) has been used as a source for oil extraction and production of textile fibers, as well for human nutrition or therapeutic use. In particular, the biggest producers of flax seeds (FS) are Canada, Argentina, India, China and New Zealand, while in Europe flax seeds are cultivated in France, Britain and Belgium. However, in the central and southern Italy there are 11 different FS cultivars [3]. In recent years, FS have attracted considerable interest for its potential health benefits, including the prevention of chronic non-communicable diseases, the cardiovascular disease reduction, atherosclerosis, diabetes, cancer, arthritis, osteoporosis, and neurological disorders [4,5,6]. In particular, different studies have reported that the FS components are effective in reducing breast cancer risk and tumour growth, or in interacting beneficially with breast cancer drugs [7]. However, the interest in this type of cancer is great because breast cancer is one of the most commonly diagnosed cancers (1.67 million cases worldwide) [8]. In particular, it is usually classified according to the expression of estrogen receptors (ER), progesterone receptors (PR), or human epidermal growth factor receptors (HER2) [9].

In different *in vivo* rodent models of breast cancer, a diet rich in FS, containing high amounts of lignans, resulted in an effective reduction of tumour growth [10]. Observational *“in vivo”* studies on post-menopausal women demonstrated that the intake of FS changes the sex hormone levels in the urine and/or serum [11]. Furthermore, some clinical trials have highlighted that FS reduce the tumour growth in breast cancer patients by means of the decrease of both cell proliferation and angiogenesis as well as by increasing apoptosis through the modulation of the estrogen metabolism and the signaling pathway of the growth-factor receptor [11]. However, recently it has been also reported that FS oil enhanced the effectiveness of trastuzumab in reducing the growth of BT-474 HER2-overexpressing human breast tumours [7]. Moreover, stearidonic acid-enriched FS oil was found to reduce the growth of human breast cancer *“in vitro”* as well as *“in vivo”* [10]. In 2010, another “*in vivo*” study determined the effect of ALA-rich oil (FSO) on the growth of estrogen receptor-positive human breast tumours (MCF7) in ovaryectomized athymic mice with high premenopausal-like estrogen (E2) levels [12]. Compared to controls, FSO showed a reduction of the tumour size and of the tumour cell proliferation, with increased apoptosis [12]. Saggar *et al*. determined the effect of SDG and alpha-linolenic acid-rich oil (FO), alone or in combination, on the growth of MCF-7 cells evidencing that SDG had a main effect in the reduction of PS2, BCL2, and IGF-1R mRNA expression, whereas FO had a main effect only in PAKT reduction [13].

Since no detailed information was previously reported concerning the effect of the phenols in FS oil on breast cancer, our aim was to characterize the phenolic extract from FS oil, and to assess its potential effects on two human breast cancer cell lines, MCF7 and MDA-MB231, and on the human non-cancerous breast cell line, MCF10A, in terms of cell growth, cell death, cell cycle, cell signaling, and lipid peroxidation. Moreover, we have also evaluated and correlated by a network analysis the expression of some genes known for their pro-oxidant properties. Surprisingly, the results show that the extract acts as a metabolic probe highlighting the differences between the two different cell types of breast cancer.

## 2. Results and Discussion 

### 2.1. Characterization of the Phenolic Extract from FS Oil

The phenolic extract from FS oil was prepared as reported in the Experimental section. At first, a screening of different solvent extractions has been performed to evaluate the efficiency of the extraction method for the bioactive compounds. In our experiments, the yield of phenolic compounds (TPC) extracted by 80% ethanol (12.3 ± 1.13 mg GAE/100 g oil: expressed as gallic acid equivalents of FS oil) resulted significantly higher in comparison to that obtained from other solvents. In fact, many studies have demonstrated that aqueous solutions of ethanol are the most effective solvents to extract phenolic compounds from various plant constituents [14,15,16]. However, our TPC value is lower than that present in extracts from murta leaves (about 50 mg GAE/100 g oil) [17] and in FS extracts obtained through different solvent extractions (that ranged from 1360 mg to 3260 mg GAE/100 g) [18].

On the other hand, if we express the total phenolic content as caffeic acid equivalents (mg CAE/100 g), we obtain a value of 4.6 mg CAE/100 g for the total phenolic content (TPC). This value is higher than that reported by Siger *et al*. [19], who analyzed the total phenolic content of various seed oils including FS, and returning a value of 1.14 mg CAE/100 g.

**Figure 1:**
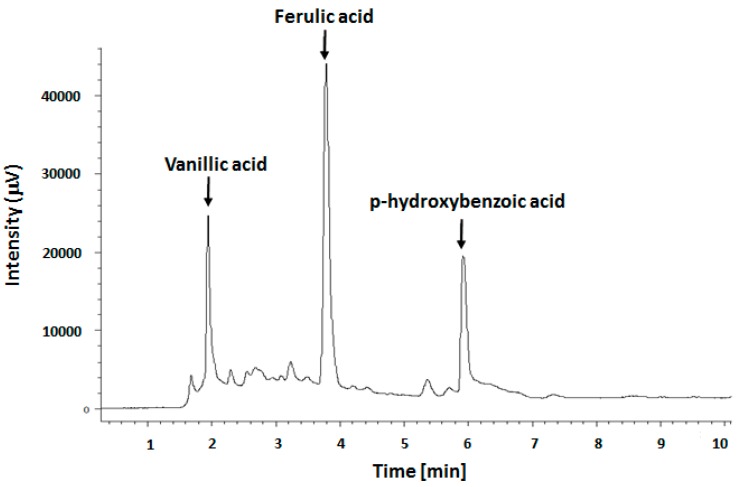
RP-HPLC separation of phenolic compounds for our phenolic extract from FS oil.

Figure 1 shows the RP-HPLC chromatogram of our phenolic extract, characterized by the presence of three major peaks assigned to phenols: ferulic, vanillic and *p*-hydroxybenzoic acids (Table 1 and Figure S1) by their respective molecular markers.

To compare the amounts of these three phenols in our extract with those found by other researchers, we expressed their amounts also as µg/100 g of oil, and obtained for ferulic, vanillic and *p*-hydroxybenzoic acids the following values: 4.1 ± 0.05, 1.3 ± 0.1 and 2.7 ± 0.09 µg/100 g of oil, respectively. Our amounts are slightly different from those found by Siger *et al*., [19], who, instead, found greater amounts of *p*-hydroxybenzoic acid (3.1 ± 0.07 µg/100 g of oil) but lower values of ferulic and vanillic acids, in the amount of 1.0 ± 0.05 and 1.0 ± 0.15 µg/100 g of oil, respectively.

Undoubtedly, it is important to underline that variations of the amount of the extracted phenols, as observed in the various papers, may safely be attributed to various factors, such as different cultivars, or modified pedogenesis processes by different soil characteristics and climatic conditions, as well as for different extracting techniques, or conditions, such as solvents and temperature.

We evaluated also the antioxidant activity of the phenolic extract by FRAP and DPPH assays that resulted equal to 0.39 mmol Fe(II)/kg of extract and 21.1%, respectively. Concerning the DPPH radical scavenging capacity, we can underline that the value obtained for our extract is higher than that reported for flaxseeds (19.3% ± 2.1%) [19], and lower than those reported for extracts from murta leaves (19.96% ± 0.370%) [20], for extracts from flaxseed hull (49.5% ± 8.23%) [17] and for FS extracts through different solvent extractions (that ranged from 42.2% ± 0.6% to 87.5% ± 1.0%) [18]. Certainly, these data depend on the TPC present in our extract.

### 2.2. Cytotoxic Effect of the Phenolic Extract from FS Oil

**Figure 2:**
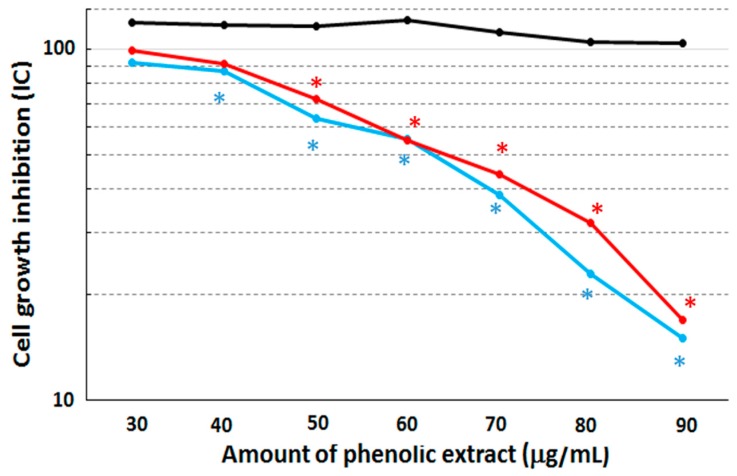
Cytotoxicity assay. We show the cell growth inhibition after 48 h of treatment with different amount of phenolic extract from FS oil on normal human breast epithelial cells, MCF-10A (in black), and two human breast cancer cells, MCF7 (in cyan) and MDA-MB231 (in red). The statistically significant differences between treated and untreated samples are indicated by *.

Cytotoxicity assays were performed on the MCF10A, MCF7 and MDA-MB231 cell lines after 48 h of stimulation with the phenolic extract by means of the colorimetric SRB assay (Figure 2).

After this treatment, both the MCF7 and MDA-MB231 cell lines displayed a cell growth inhibition corresponding to an IC_50_ concentration equal to 63 µg/mL and 64.5 µg/mL, respectively, when compared to non-treated cells. The cytotoxic effect become statistically different (with *p*-value = 0.039 and 0.045, respectively) between treated and untreated cells at the concentration of 40 µg/mL in both MCF7 and MDA-MB231 cell lines. On the other hand, the MCF10A cell line retained a quite constant viability with increasing concentrations of the extract, suggesting that our extract shows no growth effects over the assay duration. Therefore, we can conclude that the phenolic extract is able to induce macroscopically similar cytotoxicity effects on the two cancerous cell lines.

### 2.3. Apoptosis Increase after Treatment with the Phenolic Extract from FS Oil

To analyze the apoptotic contribution to this process, we have treated the MCF7 and MDA-MB231 cells with the phenolic extract from FS oil at the corresponding IC_50_ concentrations of 63 and 64.5 µg/mL, respectively. After 48 h of treatment, the MCF7 cell line showed an increase of the apoptotic cells of about 62% compared the percentages evaluated in the treated and untreated cells, whereas the percentage of living cells decreased from 74.95% to 17.30% (Table 2).

Although the MDA-MB231 cell line also showed an increase of the percentage of apoptosis, it was low enough (only from 9.71% to 22.95%) (Table 2) thus evidencing that the decreased cell proliferation was followed by a cell death increase in MCF7, but not in MDA-MB231 cells. This suggests that for the MDA-MB231 cell line, the treatment may induce an alteration of the cell cycle, as well as the putative activation of different biological mechanisms.

However, it is important to highlight that this differentiated response certainly depends by intrinsic metabolic differences between the two breast cancer cell lines. Indeed, the MCF7 cells are estrogen-receptor-positive whereas the MDA-MB231 cells are estrogen-receptor-negative. Moreover, MDA-MB231 cells are known to constitutively express the mutated p53 gene, whereas the MCF7 cells have the wild-type p53; therefore, the MDA-MB231 cell line is characterized by a more malignant phenotype [21,22]. Furthermore, as no IC_50_ value was detectable for the MCF10A cell line (see Figure 2), we decided to treat these cells at the concentration of 64.5 µg/mL, corresponding to the highest value found for IC_50_. Results show that the percentage of living cells decreased only from 92.30% to 87.50%, thus evidencing that the treatment with the phenolic extract did not induce cell death in the human non-cancerous breast cell line, MCF10A.

We are performing further studies to confirm these results by western blotting analysis. Preliminary data show a PARP-1 cleavage induction in MCF7 cells after treatment with 63 µg/mL of phenolic extract confirming the apoptosis activation (Figure S2).

### 2.4. Effect of the Phenolic Extract from FS Oil on Cell Cycle

Considering that the phenolic extract affected the cell proliferation in both cell lines inducing death in MCF7 but not in MDA-MB231 cells, we analyzed its effect on the cell cycle after 48 h of treatment at the same concentrations previously used for the apoptosis studies on MCF7, MDA-MB231 and MCF10A cells. Our results show that no visible change was detected for the MCF10A cell line.

On the other hand, we observe a cell cycle population shift and an increase of cells in the G0/G1 phase mainly for the MCF7 cell line (Table 3). In particular, in the cell cycle of MCF7 we also observed an accumulation of apoptotic cells in the subG1 region after 48 h of exposure, and this is in good agreement with the apoptosis experiments reported above.

All these results have evidenced that this extract acts in a different way on the cell cycles of the two breast cancer cell lines and this can be reasonably correlated to the different effect on the apoptosis inducted by the phenolic extract on MCF7 and MDA-MB231 cells.

### 2.5. Modulation of H2AX and PI3K Pathways after the Treatment with the Phenolic Extract from FS Oil

To gain a greater understanding of the metabolic effects of the phenolic extract on these cell lines, we focused on two critical signaling molecules, H2AX and PI3K. In particular, H2AX is a nucleoplasmatic histone required for checkpoint-mediated arrest of cell cycle progression in response to DNA double strand breaks specifically when modified by C-terminal phosphorylation on serine 139 [23] whereas PI3K plays a key role by activating signaling cascades involved in cell growth, survival, and proliferation.

In details, we have investigated the ability of the phenolic extract in modulating the activation of H2AX and PI3K pathways in MCF7, MDA-MB231 and MCF10A cells after 48 h of cell treatment using the same concentrations used for apoptosis and cell cycle evaluation. For this purpose, we have determined the percentage of activated cell population, compared to the total cell population. Regarding the activation of H2AX, after stimulation with the extract we have observed an evident and statistically significant activation, evaluated between un-treated and treated cells by T-test (with *p*-value = 0.041), only for the MCF7 cell line (42%), when compared to untreated cells used as control, whereas the activation for the MDA-MB231 and MCF10A cell lines was equal only to about 4% (Table 4). Some studies have evidenced that anticancer treatment may lead to an increase of H2AX phosphorylation due to death-associated DNA fragmentation, especially after treatment with drugs suggesting the presence of relationships between chromatin architecture, response to DNA damage stimulus, regulation of DNA repair and apoptosis [23,24]. Therefore, we can conclude that the specific activation of H2AX, found in the MCF7 cells after treatment with our extract, is correlated to the increase of the apoptosis.

On the contrary, after treatment with the phenolic extract, the PI3K pathway showed no significant activation in MCF7 (1%), as well as in MCF10A (1.5%) cell line, when compared to controls, while a small but statistically significant activation (16%), evaluated between untreated and treated cells by T-test (with *p*-value = 0.047), was found in MDA-MB231 cells (Table 4). It is well known that the PI3K-Akt signaling pathway is activated by many types of cellular stimuli or toxic insults and is indicated as an important survival pathway [25], where PI3K is a lipid kinase that generates PI-3,4,5-tris phosphate (PIP3), a second messenger essential for the Akt translocation to the plasma membrane. In fact, PI3K is indicated as an anti-apoptotic effector of the growth factor signaling pathway [26], and the activation of the PI3K-Akt pathway has been reported to reduce the apoptosis of tumor cells [27]. Therefore, we can conclude that the lack of activation of PI3K signaling in MCF7 cells after treatment is strictly correlated to the cell death whereas the poor activation in MDA-MB231 cell line is linked to their cell survival. 

### 2.6. Increase of the Lipid Peroxidation after the Treatment with the Phenolic Extract from FS Oil

Lipid peroxidation is one of the most investigated consequences of reactive oxygen species (ROS) actions on the membrane structure and function. It has been shown that lipid hydroperoxides can participate in the signal transduction cascade, in the control of cell proliferation, and the induction of differentiation, maturation, and apoptosis [28]. Indeed, both lipid peroxidation and ROS are essential apoptosis mediators, devoted to eliminate pre-cancerous and cancerous, virus-infected or otherwise damaged cells [29]. However, the aldehydes generated from lipid peroxidation form DNA adducts and lipid hydro-peroxides inducing the extensive single and double strand breaks [30], which activate a variant of histone H2A, at Ser139. Hence, the membrane lipid peroxidation and DNA double-strand breaks are considered to be manifestations of the oxidative cell damage.

**Figure 3:**
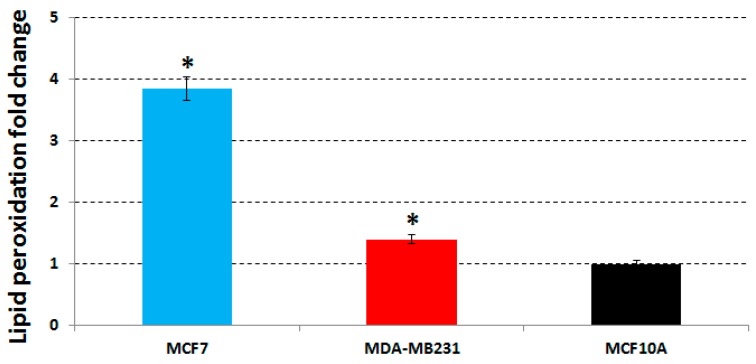
Evaluation of the lipid peroxidation. We show the changes in the expression levels of lipid peroxidation of MCF7 (in cyan), MDA-MB231 (in red) and MCF10A (in black) cell lines treated at concentrations of 63 µg/mL, 64.5 µg/mL and 64.5 µg/mL of phenolic extract compared to those of untreated cells. Data are reported as the mean value plus/minus the standard deviation. Statistically significant differences between treated and untreated samples are indicated by *.

The lipid peroxidation levels in MCF7, MDA-MB231 and MCF10A cell lines treated with the same concentrations used in the previous assays, were determined by measuring the amount of the first product of lipid peroxidation, *i.e.*, peroxides, in untreated and treated cells. We found that a 48 h treatment induced: (1) an increase of the lipid peroxides production in MCF7 cells with a fold change of 3.85 compared to untreated cells; (2) a weak increase of the lipid peroxides in MDA-MB231 cells with a 1.4-fold change; and (3) no lipid peroxide increase was found for MCF10A cells (Figure 3). Therefore, we can conclude that the extract acts on MCF7 cells by inducing oxidation, and that this pro-oxidant effect of the phenolic extract is strictly correlated with the activation of H2AX (Table 4) as well as with the apoptosis increase (Table 2) found always in MCF7 cells.

### 2.7. Gene Expression Evaluation after the Treatment with the Phenolic Extract from FS Oil and Network Analysis

To deepen our observations on the mode of action of our extract, we have evaluated the expression levels of IL-1β, IL-6, of the two subunits of the nuclear factor kappa-light-chain-enhancer of activated B cells (NF-κB) complex, NFKB1 and RELA, iNOS, cytochrome P450 family 1, subfamily A polypeptide 1 (CYP1A1) and glutathione peroxidases (GPXs 1-7) for MCF7, MDA-MB231 and MCF10A cell lines, treated with the same concentrations of phenolic extract used in the previous assay. The expression levels were evaluated by RT-qPCR, and compared with the untreated cells.

The choice of these specific molecules has been dictated by the fact that they represent key points of control at various levels, *i.e.*, extracellular, cytoplasmic, and nuclear.

**Figure 4:**
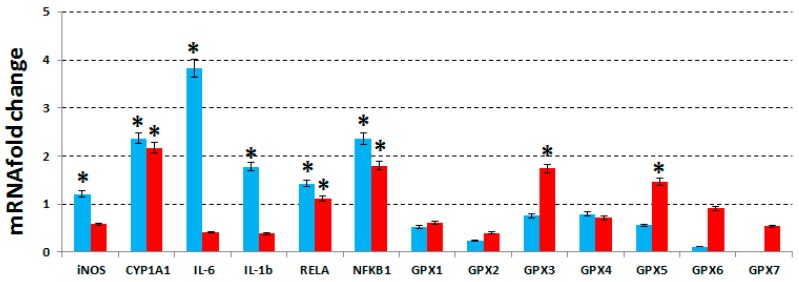
RT-qPCR analysis. We show the fold changes evaluated as ratio between the expression levels of 13 genes in two breast cancer cell lines, MCF7 (**cyan**) and MDA-MB231 (**red**), treated with 63 g and 64.5 g of phenolic extract from FS oil, compared to those in the untreated cells. We report the fold changes as mean value ± standard deviation. The statistically significant differences between treated and untreated samples are indicated by *.

As shown in Figure 4, in MCF7 cells, IL-1β, IL-6, two subunits of NF-κB, iNOS and CYP1A1 resulted to be up-regulated after treatment, while in MDA-MB231 cells, NF-κB subunits and CYP1A1 were found up-regulated and IL-1β, IL-6 and iNOS were unaffected.

Even GPXs from 1 to 7 has been proven to be unchanged in MCF7 cells but GPX-3 and -5 were found upregulated in MDA-MB231. No change was observed in MCF10A cells (data not shown). In the literature it is reported that the NF-κB activation either promotes or blocks the apoptotic cell death, depending upon the cell type and the nature of the oxidative stimuli [31]. In estrogen-receptor-negative MDA-MB231 cells, NF-κB is constitutively activated contributing to the survival signals that is also responsible for resistance to treatments [32]. Although the expression of NF-κB target genes in most events promotes cell survival, and has, hence, an anti-apoptotic effect; nevertheless, there are some exceptions when NF-κB activation may lead to cell death, acting as an apoptotic gene regulator [33]. In these cases, NF-κB is regulated by pro-inflammatory cytokines, such as IL-6 and IL-1β, and in general terms, NF-κB traditionally refers to the NFKB1/RELA (p50/p65) heterodimer. Ricca *et al*. [34] demonstrated that the upregulation of RELA in MCF7/ADR breast cancer cells led to robust cell death, implying a direct pro-apoptotic role for NF- kB in these cell lines. In fact, RELA alone has been found to be adequate for inducing cell death [35]. Therefore, the upregulation of NF-κB after treatment with phenolic extract in MCF7 cells is strictly correlated to the expression increase of IL-1 and IL-6, and, also, to the apoptosis increase and the cell cycle arrest (Table 2 and Table 3). On the other hand, also the upregulation of iNOS is determined by IL-1 and IL-6, since the expression of these pro-inflammatory cytokines is typically missing in unstimulated cells [36]. However, previous studies have also shown that nitric oxide (NO) is an important modulator of apoptosis in islet cells, and that the rapid induction of iNOS expression can trigger NO-dependent apoptosis “in vitro”, as a consequence of DNA damage and that it may be mediated by a p53-dependent apoptotic pathway [37].

Hence, IL-1, IL-6, NF-κB subunits and iNOS are correlated with each other and this result can explain why all these five genes are up-regulated in MCF7 cells. Furthermore, among the genes involved in stress and toxicity pathways, we have evaluated the expression of CYP1A1, which plays an important role in the oxidative metabolism, catalyzes many reactions involved in drug metabolism and synthesis of lipids and estrogens, and results to be modulated in cancer cell lines after their treatment with phenols [38].

Finally, we have also evaluated the expression of GPXs 1-7, which were unchanged in both MCF7 and MCF10A cells (Figure 4). In general, the GPXs are an enzyme family with peroxidase activity whose main biological role is to protect the organism from oxidative damage. Their specific biochemical function is that of reducing the lipid hydro-peroxides to their corresponding alcohols and the free hydrogen peroxide to water [39]. Therefore, their unchanged expression in MCF7 cells could depend on the high levels of lipid peroxidation found in MCF7 cells after treatment, whereas the up-regulation of two GPXs could explain the low levels of lipid peroxidation found in MDA-MB231 cells.

**Figure 5:**
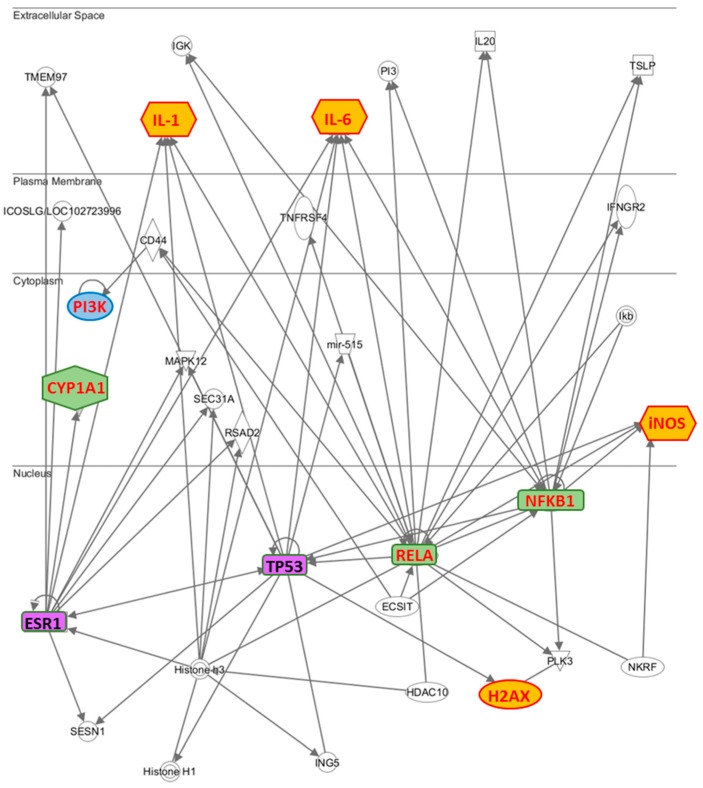
Interactomic analysis by Ingenuity Pathway Analysis (IPA) of significant molecules. The interactome shows the close functional association between the molecules evaluated in this work as well as the paths in which other functionally relevant molecules are also involved. In details, we report in yellow the genes modulated only in MCF7 cells like three up-regulated genes (IL-1, IL-6 and iNOS) and the activated H2AX, in cyan PI3K that is activated only in MDA-MB231 cells, in green the genes (CYP1A1, NFKB1 and RELA) that are up-regulated in both cell lines, and in pink the other two HUB genes, TP53 and ESR1.

To highlight which relationships were present between the molecules examined by us, we have performed an interactomic analysis. This kind of analysis is performed on the basis of known associated functions and data mining from experimental studies reported in the literature. The results confirm that the most part of the analyzed molecules is involved in an extensive network (Figure 5). Further, the network reveals that some proteins are connected through some HUB nodes such as NF-κB subunits (NFKB1 and RELA), TP53 (tumor suppressor 53) and ESR1 (estrogen receptor 1), which is commonly used as clinical breast cancer marker. More specifically: (i) IL-1 and IL-6 are connected with ESR1, NFKB1, RELA and TP53, CYP1A1 with ESR1, H2AX with TP53, PI3K with RELA through CD44 and iNOS with NFKB1, RELA and TP53.

While on the one hand, these data evidence the crucial role developed by TP53 and ESR1, on the other hand how they are correlated with NFKB subunits, demonstrating why the two breast cancer lines, MCF7 and MDA-MB231, respond in different way to the treatment with the phenolic extract. In fact, since the MDA-MB231 cells are known to constitutively express the mutated p53 gene [21,22], TP53 is not able to modulate the activation of IL-1, IL-6, H2AX, and iNOS in these cells, explaining why these genes are not modulated by phenolic extract.

## 3. Experimental Section

### 3.1. Chemicals and Reagents

All HPLC standards, Folin-Ciocalteu’s phenol regent (2 N), 2,2-diphenyl-1-picrylhydrazyl (DPPH) were obtained from Sigma-Aldrich (St. Louis, MO, USA). All other chemicals/reagents and solvents used in this study were of analytical grade and purchased from Merck (Darmstadt, Germany). The flaxseeds derived from the commercial cultivar Barbara grown in the Umbria Region (Italy). In our cold-pressing extraction procedure we used crude oil from FS, where pure and clear oil has been pressed out of the seeds at room temperature. In details, the crude oil has been used for our experiments in less of fifteen days from its extraction and always stored in opaque bottle under pure nitrogen at 6 °C to preserve it from oxidation. The water used for solutions/extractions was always distilled and saturated by pure N_2_ to avoid oxidation phenomena.

### 3.2. Phenolic Compounds Extraction

Phenolic samples were extracted from 10 g of oil obtained from FS with 80% aqueous ethanol (10 mL/g of oil per extraction) by triple extraction using an Ultra-Turrax homogenizer. The ethanol extracts obtained by centrifugation (2 min, 5000 *g*) were concentrated to dryness by rotary evaporation (30 °C in a water bath) and the resulting residue was stored in a freezer (−20 °C) for subsequent analyses. For HPLC analysis, 2 mL of the extract was lyophilized and concentrated to dryness under vacuum (SVC100H Speedvac Concentrator, Savant, Missouri City, TX, USA) at room temperature.

### 3.3. Total Phenolic Acids Determination

For the assay deionized water (7 mL), Folin-Ciocalteu’s reagent (0.5 mL) and phenolic extract (0.2 mL) were mixed in a 10 mL volumetric flask. After three minutes, saturated sodium carbonate and distilled water (1 mL) were added until a total volume of 10 mL. Samples were vortexed and placed in the dark for 60 min prior to analysis. The absorbance at 725 nm was determined using an UV/Vis Spectrophotometer (mod. DU 730, Beckman Coulter, Fullerton, CA, USA). Samples were quantified against the calibration curve of both gallic acid and caffeic acid (≥99% purity). In practice, the results were expressed as mg of gallic acid equivalent per kg of FS oil (mg GAE/kg oil) and also as mg of caffeic acid equivalent per kg of FS oil (mg CA/kg oil). Samples were assayed in triplicate and values were averaged. 

### 3.4. Phenolic Acid Composition by HPLC Analysis

The phenolic components separation was performed by LC-4000 Series Integrated HPLC Systems (JASCO, Japan) Lyophilized phenolic extracts were dissolved in 1 mL of methanol and then filtered through Whatman filter paper. Then, 20 µL of sample were loaded on a C18 Hypersil Gold (Thermo Fisher Scientific Italia, Rodano, Milano, Italy ) column and eluted at 1 mL/min by means of a gradient program (0–35 min, 10% B; 35–40 min, 55% B; 40–45 min, 100% B; 45–50 min, 10% B) using a solution of 2% acetic acid as solvent A and 0.5% acetic acid in 50% acetonitrile as solvent B. UV detection was carried out at 280 nm. Phenolic compounds were identified by comparing their retention times with those of pure standards. Results were expressed as µg of phenolic compound/100 g of FS oil.

### 3.5. Antioxidant Activity

The antioxidant activity of the phenolic extract was determined by the FRAP assay, according to Szöllösi and Szöllösi-Varga [40]. Dry extract was resuspended in 200 μL of ultrapure water. To 50 μL of sample, 1.5 mL of freshly prepared FRAP reagent (50 mL of 300 mM acetate buffer, pH 3.6; 5 mL of 10 mmol TPTZ in 40 mM HCl; 5 mL of 20 mM FeCl_3_·6H_2_O) were added. The absorbance was recorded at 593 nm after 5 min. The antioxidant activity was expressed as mmol Fe (II)/kg of extract.

Moreover, the free radical scavenging capacity was determined by the DPPH assay [41]. An aliquot of 500 L of sample solution in ethanol was mixed with 500 L of 0.5 mM DPPH in ethanol. The mixture was shaken vigorously and incubated for 30 min at room temperature in the dark. Ethanol was used as the blank. The absorbance was measured at 517 nm. The antiradical activity was expressed as percentage of inhibition (I%) of the sample (As) compared to the initial concentration of DPPH (Ac) according to the formula:

I% = (Ac − As/Ac) × 100
(1)

### 3.6. Cell Culture and Treatment

Two human breast cancer cell lines, estrogen-receptor-positive MCF7 (HTB-22, adenocarcinoma) (Lonza, Verviers, Belgium) and estrogen-receptor-negative MDA-MB231 (HTB-26, adenocarcinoma) (Lonza), and the human non-cancerous breast cell line, MCF10A (CRL-10317, fibrocystic disease) (Lonza) were kept in culture. In details, MCF7 and MCF10A were expanded at 37 °C in a humidified atmosphere of 5% CO_2_ in culture medium DMEM (Dulbecco’s Modified Eagle’s Medium, Lonza), whereas MDA-MB231 in RPMI 1640 (Lonza), supplemented with FBS (Invitrogen, Camarillo, CA, USA) at 10%, Penicillin/Streptomycin 100× (Euroclone, Devon, UK), Glutamax 100× (Invitrogen) non-essential amino acids 100× (Invitrogen). Moreover, in the case of MCF10A the DMEM was supplemented also with human insulin 10 μg/mL (Life Technologies Corporation, Carlsbad, CA, USA), human epidermal growth factor 20 ng/mL (Life Technologies), and hydrocortisone 0.5 μg/mL (Sigma-Aldrich) according to the procedure reported in Rothwell *et al*. [42]. We know that the presence of insulin and hydrocortisone, which are required for culturing of MCF10 cells, can have effect on antioxidant capacity of cells, therefore we have used these cells because they represent one of the internationally models used for these types of studies. Phosphate buffer (PBS phosphate buffered saline Ca^2+^ and Mg^2+^ free) and trypsin (Ca^2+^ and Mg^2+^ free) were supplied by Euroclone. The cells were plated 25 × 10^3^ for well in 96 well tissue culture plates and left to grow for 24 h at 37 °C to allow adhesion. The experiments started at 80% cell confluence. Then, the cells were treated with varying concentrations of phenolic extract from FS oil at 30, 40, 50, 60, 70, 80, 90 µg/mL and incubated for 48 h in according to a procedure already used in our papers [43,44]. These concentrations were selected on the basis of previous results on the effect of *Linum usitatissimum* extracts on human breast cancer cell lines [45]. The phenolic extract from FS oil was dissolved in dimethyl sulfoxide (DMSO 100 mM, Sigma-Aldrich). In cell cultures the DMSO concentration remained always below 0.1%, a dose that did not exert toxic effects [46]. In fact, we prepared a stock solution (100 mg/mL) and serial dilutions were made to obtain the different amounts of extracts (reported above) with a final concentration of 0.05% DMSO.

### 3.7. Sulforhodamine B Assay

After 48 h of exposition to phenolic extract, the cell proliferation was measured by a spectrophotometric assay, which incorporated sulforhodamine B (SRB) as dye. The sulforhodamine B test is a colorimetric test that shows the cell proliferative behavior subjected to the action of the tested substances [47]. Cells were fixed with trichloroacetic acid (Sigma-Aldrich) for 1h and after stained for 30 min with 0.4% (*w/v*) SRB (Sigma-Aldrich) dissolved in 1% acetic acid. The viable cells number was directly proportional to the protein bound-dye formation which was then solubilized with 10 mM Tris base solution pH 10.5 and measured by fluorometric assay ELISA at 540 nm on a microplate reader (Bio-Rad, Hercules, CA, USA). All experiments were performed in triplicate and were repeated for three times. Cellular viability was estimated as % compared to untreated cells. In details, the cell growth inhibition after 48 h treatment was evaluated as the ratio between [the average value of the absorbance at 540 nm obtained in treated and untreated cells] × 100. The IC_50_ was assessed from the dose-response curves.

### 3.8. Apoptosis Detection

We counted and analyzed 3 × 10^5^ cells for the apoptosis detection assay. These cells were harvested and washed twice with ice-cold PBS. Subsequently, the cells were labeled with “Annexin V & Dead Cell Assay kit” according to the manufacturer’s instructions (Merck Millipore, Darmstadt, Germany). This assay is based on the phosphatidylserine (PS) detection on the apoptotic cells surface, using fluorescently labelled Annexin V in combination with the dead cell marker, 7-aminoactinomycin D (7-AAD). We have calculated the apoptotic ratio by identifying four populations: (1) the viable cells, not undergoing detectable apoptosis: Annexin V (−) and dead cell marker (−); (2) the early apoptotic cells: Annexin V (+) and dead cell marker (−); (3) the late apoptotic cells: Annexin V (+) and dead cell marker (+); and (4) the cells died through non-apoptotic pathway: Annexin V (−) and dead cell marker (+). The samples were counted by the Muse™ Cell Analyzer (Merck Millipore) and analyzed by a software provided by Merck Millipore. 

### 3.9. Protein Extraction and Western Blotting

Cells were washed once in cold phosphate buffered saline (PBS) and lysed in a lysis buffer containing 50 mM *N*-(2-hydroxyethyl)-piperazine-*N*-2-ethanesulfonic acid, 150 mM NaCl, 1 mM ethylene-diamine tetraacetic acid, 1 mM ethylene glycol tetraacetic acid, 10% glycerol, 1% Triton-X-100, 1 mm phenyl-methyl-sulfonyl fluoride, 1 g aprotinin, 0.5 mm sodium orthovanadate and 20 mm sodium pyrophosphate. The lysates were clarified by centrifugation at 16,000 *g* for 10 min. Protein concentrations were estimated by a BioRad assay (BioRad) and boiled in Laemmli buffer (Tris-HCl 0.125 m pH 6.8, sodium dodecyl sulphate (SDS) 4%, glycerol 20%, 2-mercaptoethanol 10%, bromophenol blue 0.002%) for 5 min before electrophoresis. Proteins were subjected to SDS-polyacrylamide gel electrophoresis (PAGE) (15% polyacrylamide) under reducing condition. After electrophoresis, proteins were transferred to nitrocellulose membranes (Immobilon-P Millipore Corp., Bedford, MA, USA). The complete transfer was assessed using pre-stained protein standards (BioRad). After blocking with Tris-buffered saline (TBS)-bovine serum albumin (BSA) (25 mm Tris, pH 7.4, 200 mm NaCl, 5% BSA). The membranes were incubated with the specific primary anti-human antibody PARP-1 1:500 (Santa Cruz Biotechnology, Inc., Dallas, TX, USA) overnight at 4 °C. When the membranes were washed and incubated with anti-rabbit horseradish peroxidase conjugate at a dilution of 1:3000 for 1 h at room temperature. The immune-reactive bands of proteins were visualized by enhanced chemiluminescence immunoassay method (ECL Amersham Biosciences, Little Chalfont, UK). The blots were stripped and re-probed with anti-GAPDH antibody (Santa Cruz Biotechnology) to normalize for differences in protein loading.

### 3.10. Cell Cycle Assay

The Muse™ Cell Cycle Assay uses a premixed reagent containing the nuclear DNA intercalating stain propidium iodide (PI) and RNAse A in a proprietary formulation. PI discriminates cells at different stages of the cell cycle, based on the differential DNA content in the presence of RNAse to increase the specificity of DNA staining. The samples were centrifuged at 300× *g* for 5 min and after removing and discarding the supernatant, an appropriate volume of PBS was added to each tube (1 mL of PBS per 1 × 10^6^ cells). After centrifugation and removing of the supernatant, 1 mL of ice cold 70% ethanol was added to the re-suspending cell pellet in the residual PBS. The tubes were capped and frozen at −20 °C for at least 3 h prior to staining. Ethanol-fixed cells were centrifuged at 300× *g* for 5 min at room temperature and the pellet was re-suspended in PBS. The cells were centrifuged again at 300× *g* for 5 min at room temperature, the supernatant was removed and discarded and cell pellet was re-suspended in 200 μL of Muse™ Cell Cycle Reagent and incubated for 30 min at room temperature, in the dark. Cell suspension samples were transferred to a 1.5 mL micro-centrifuge tubes prior to analysis.

### 3.11. Cell Signaling Pathways Analysis

After 48 h, the cells (treated and untreated) were centrifuged at 300× *g* for 5 min and suspended by adding 500 µL of 1× Assay Buffer and 500 µL of Fixation Buffer for one million cells (1:1). The cells were incubated for 5 min on ice. After spinned down at 300× *g* for 5 min, the cells were permeabilized by adding 1 mL ice-cold Pemeabilization Buffer and incubated on ice for 5 min. The cells were centrifuged and suspended in 450 µL 1× Assay Buffer. Then the cells were incubated with 10 µL of antibody (anti-H2AX and PI3K) for 30 min in the dark at room temperature. After that the cells were suspended in 100 µL of 1× Assay Buffer and were centrifuged, they were suspended in 200 µL of 1× Assay Buffer ad acquired on the Muse Cell Analyzer. The Muse^®^ H2AX Activation Dual Detection Kit includes two directly conjugated antibodies, a phospho-specific anti-phospho-Histone H2AX (Ser139)-Alexa Fluor 555 and an anti-Histone H2AX-PECy5 conjugated antibody to measure total levels of Histone H2AX. The Muse^®^ PI3K Activation Dual Detection Kit includes two directly conjugated antibodies, a phospho-specific anti-phospho-Akt (Ser473), Alexa Fluor^®^555 and an anti-Akt, PECy5 conjugated antibody to measure total levels of Akt. These two color kits are designed to measure the extent of H2AX phosphorylation relative to the total H2AX expression and of Akt phosphorylation relative to the total Akt expression in any given cell population. By doing such, the levels of both total and phosphorylated protein can be measured simultaneously in the same cell, resulting in a normalized and accurate measurement of H2AX and PI3K activation after stimulation.

### 3.12. Lipocell Assay

This method allowed us to analyze the phenolic extract effect from FS oil on lipid peroxidation. It was based on the ability of peroxides to promote the oxidation of Fe^2+^ to Fe^3+^. Fe product binds to thiocyanate developing a colored complex with measurable with photometer Free Carpe Diem (Diacron s.r.l. Grosseto, Italy). The absorbance increase was directly proportional to the concentration of the peroxides present in the sample. Then, the cells were sonicated and washed with distilled water, and the absorbance was read at a 505 nm wavelength according to the manual provided by the manufacturer. The number of hydroperoxides was calculated according to the following formulas: Abs Sample/Abs Standard × 10 = nanomoles of hydroperoxides contained in the sample nanomoles of hydroperoxides contained in the sample/number of cells used × 2 = nanomoles of hydroperoxides million cells.

### 3.13. RNA Preparation and Reverse Transcription-qPCR (RT-qPCR) Analysis 

Total RNA was extracted from MCF10A, MCF7 and MDA-MB231 cell lines using the RNAeasy mini kit (Qiagen Inc., Valencia, CA, USA) according to the manufacturer’s instructions. The extracted RNA was dissolved in diethyl pyrocarbonate treated water, and its concentration and purity were assessed by measurement of optical density at 260/280 nm. RNA samples were quantified using a NanoDrop 2000 spectrophotometer (Thermo Scientific, Wilmington, DE, USA). Two microgram of total RNA of each sample was reverse-transcribed with SuperScript VILO cDNA Synthesis kit (Life Technologies) according to the manufacturer’s instructions and subsequently diluted with nuclease-free water (Life Technologies-Ambion). Sequence for mRNA from the nucleotide data bank (National Center for Biotechnology Information, Bethesda, Maryland, USA) was used to design primer pairs for RT-qPCR (Primer Express, Applied Biosystems, Lincoln Centre Drive Foster City, CA, USA). Oligonucleotides were obtained from Sigma Aldrich. The primer sequences are provided in Table 5.

An appropriate region of 18S rRNA was used as control. RT-qPCR assays were run on a Step-One Real Time PCR System (Applied Biosystems). cDNA (2 µL) were amplified in a total volume of 25 µL containing 2× SYBR Green PCR Master Mix (Applied Biosystems) and 300 nM of forward and reverse primers. The thermal cycling conditions were as follows: 5 min of denaturation at 95 °C followed by 44 cycles of a two-step program (denaturation at 95 °C for 30 s and annealing/extension at 60 °C for 1 min). Dilutions of standards and test samples were run in duplicate. Each reaction was repeated at least three times. Data were normalized using the 18S rRNA as housekeeping gene which has been previously used in our recent paper [48].

### 3.14. Statistical Analysis

All the data are reported as mean values plus/minus standard deviation. The differences of levels between un-treated and treated cells were evaluated by *t-*test assay (GraphPad software, La Jolla, CA, USA). Significance was defined as *p* < 0.05 and indicated with one asterisk (*). In RT-qPCR analysis, the expression levels of each target gene in treated cells were compared with those untreated; thus, the fold change was calculated as ratio between levels of treated and un-treated cells. The 1×-fold expression level was chosen as the threshold for the significance of target genes. Moreover, a network analysis was performed between the most significant proteins by Ingenuity Pathway Analysis (IPA).

## 4. Conclusions

Since not many data are reported in the literature about the phenolic extract from FS oil, and its effects on the breast cancer, in this paper we have characterized the phenolic component extracted from this oil. Furthermore, we have assessed the effects of the phenolic extract after 48 h of treatment on two human breast cancer cell lines, MCF7 and MDA-MB231, and on the human non-cancerous breast cell line, MCF10A, by evaluating cellular growth, cellular death, cell cycle, cell signaling, lipid peroxidation and expression of some genes useful to evaluate its pro-oxidant properties. 

Our results highlighted that the phenolic extract was very effective only on the cancerous estrogen-receptor-positive MCF7 cell line by inducing: (1) a significant decrease of cell proliferation with a simultaneous increase of apoptosis and of the cell cycle G0/G1 phase when compared to untreated cells; (2) an evident activation of the H2AX signaling but not of that of PI3K; (3) an increase of lipid peroxidation; and (4) the up-regulation of CYP1A1, IL-1β, IL-6, iNOS genes, and also of genes corresponding to two subunits of NF-κB.

On the other hand, on the estrogen-receptor-negative MDA-MB2131 cells we have proven: (1) only an anti-proliferative activity; (2) a weak lipid peroxidation and the PI3K signaling pathway activation; and (3) an up-regulation of CYP1A1, of the anti-apoptotic NF-κB and of two GPXs. 

However, an important result of our study is the evident pro-oxidant effect of this phenolic extract, exerted only on the cancerous estrogen-receptor-positive MCF7 cell line through the increase of lipid peroxidation. Therefore, while this demonstrates and supports the awareness that a defensive way of these cells to fight the phenols is through the ROS production, eliminating the pre-cancerous and/or cancerous cells, which are evidently less resistant to oxidation, on the other hand this metabolic effect is practically missing in the normal cell line, MCF10A, but it is very slight on the estrogen-receptor-negative cancerous cell line, MDA-MB231. A general explanation resides on the phenotypic difference existing between the various cell lines, but at the same time also emphasizes like the two cancerous phenotypes are very different from each other, with overall responses that hide different metabolic solutions. Macroscopically, we notice that the phenotype differences of the two breast cancer cell lines, reside on the different ability to metabolize estrogens and on the different functions of the wild-type or mutated P53. Therefore, these two facts seem to affect cell fate. Concomitantly, in the MCF7 cell line we have found an increase of the pro-inflammatory cytokines, IL-6 and IL-1β, which, regulating the nuclear translocation of NF-κB necessary to mediate the expression of genes of the inflammatory response, push cells to apoptosis as well as to cell cycle arrest, in according to our results.

Therefore, a general picture that comes out from our observations suggests that two different phenotypes of breast cancer seem to respond in rather similar ways to the phenolic extract, showing in both cases an anti-proliferative activity, although in very different ways and degrees. Actually the extract acts as a metabolic crowbar, highlighting that the metabolic responses to stressful/toxic stimuli are very different. This leads to the conclusion that the phenolic extract shows specific cytotoxic and pro-oxidant role only for the cell line MCF7, through oxidative mechanisms that, altering the cell cycle, lead to an apoptotic death. Hence, in our case, one of the cancer cell lines, *i.e.*, MCF7, is metabolically different and generates a self-destructive mechanism in response to the oxidative stress induced by the phenolic extract. 

Therefore, our results agree with the impressive paper of Watson [49] that in 2013 evidenced how the most of the drugs, used to fight cancer, does not block the oxidative stress but work by producing ROS which in turn act by blocking key functions of the cell cycle and, thus, leading the cell to the death/apoptosis [49].

However, the different metabolic solutions implemented by the two lines of cancer cells require metabolic explanations experimentally not always accessible. Therefore, we used an interactomic analysis (Figure 5), based on experimental data present in the databases, to search molecular partners and metabolic functions in which they are involved. If we consider that the metabolism can be represented by a single global metabolic network including more functional levels [50], we quickly realize how are important the specific properties of nodes that make up the metabolic network. The greater is their interacting ability, the greater the number of their molecular partners. Metabolically speaking, this suggests the possible activation of a very large number of different biological functions; therefore, the possible solutions in response to an internal or external metabolic stress are different and very numerous, or better enormously numerous.

Certainly overall our data represent a preliminary study on this topic and further studies will also evaluate the effect of the phenolic extract from FS oil on breast cancer cell lines in combination with drugs commonly used during therapy. Moreover, we want also to evaluate the effect of the three main phenols on these cell lines to verify if the effect of the whole extract is due to the combined action of different phenols or to a single phenol. As one can see in Figure S3, we have already tested the effect of the ferulic acid on all the three cell lines using the same concentrations used for the extract by SRB assay. These preliminary results have evidenced that the three cell lines retained a quite constant viability with increasing concentrations of the ferulic acid, suggesting that the cytotoxicity effect on the two cancerous cell lines is not due to the presence of this single phenol.

## Figures and Tables

**Table 1:** Total phenolic content (TPC), and amounts of three main phenols fractions were evaluated from flaxseed oil. Data are the mean value of three analytical replicates and three-independent samples plus/minus the standard deviation. In details, TPC is expressed both as mg of gallic acid equivalent/100 g of oil and as mg of caffeic acid equivalent/100 g, and the three phenols as µg/g Phenolic Extract Dry Weight (DW PE).

Sample	mg GAE/100 g Oil		µg/g PE DW		
	TPC	TCP	Ferulic Acid	Vanillic Acid	*p*-Hydroxybenzoic Acids
Flaxseed oil	12.3 ± 1.13	4.6 ± 1.72	1.64 ± 0.02	0.52 ± 0.04	1.08 ± 0.04

**Table 2:** Apoptosis studies. We show the percentage of live, total apoptotic, and dead cells for MCF7 and MDA-MB231 cells before and after treatment with 63 µg/mL and 64.5 µg/mL of phenolic extract from FS oil, respectively.

Cell Line	Live	Total Apoptotic	Dead
MCF7			
un-treated	74.95 ± 0.04	20.40 ± 0.04	4.65 ± 0.3
treated	17.30 ± 0.04	82.05 ± 0.05	0.65 ± 0.03
MDA-MB231			
un-treated	90.16 ± 0.05	9.71 ± 0.04	0.13 ± 0.02
treated	75.85 ± 0.05	22.95 ± 0.04	1.20 ± 0.03

**Table 3:** Cell cycle evaluation. We show the cell percentages in G0/G1, S and G2/M phases for MCF7 and MDA-MB231 cells before and after treatment with 63 µg/mL and 64.5 µg/mL of phenolic extract from FS oil, respectively, and MCF10A with 64.5 µg/mL.

Cell Line	G0/G1 (%)	S (%)	G2/M (%)
**MCF7**			
**un-treated**	57.9 ± 2.6	20.4 ± 1.3	19.5 ± 1.4
**treated**	73.1 ± 2.1	13 ± 2.9	8 ± 2.6
**MDA-MB231**			
**un-treated**	47.4 ± 1.2	21.8 ± 1.8	29.5 ± 1.5
**treated**	57.2 ± 1.8	17.9 ± 1.9	17.8 ± 1.1
**MCF-10A**			
**un-treated**	70.3 ± 1.8	9.8 ± 2.1	19.1 ± 2.1
**treated**	83.7 ± 2.3	4.3 ± 1.8	10.5 ± 1.7

**Table 4:** H2AX and PI3K activation (expressed as percentages) for each cell population compared to the total cell population for MCF7, MDA-MB231 and MCF10A cells before and after treatment with 63 µg/mL, 64.5 µg/mL and 64.5 µg/mL of phenolic extract from FS oil. The data are reported as mean value plus/minus the standard deviation.

Cell Line	H2AX Activation Percentages	PI3K Activation Percentages
**MCF7**		
**un-treated**	11.2 ± 1.8	0.7 ± 0.8
**treated**	42.2 ± 2.3	0.9 ± 1.5
**MDA-MB231**		
**un-treated**	3.0 ± 2.1	0.6 ± 0.2
**treated**	4.1 ± 2.5	16.1 ± 0.5
**MCF10A**		
**un-treated**	8.7 ± 2.1	2.1 ± 0.2
**treated**	4.2 ± 5.0	1.5 ± 0.5

**Table 5:** Parameters for RT-qPCR analysis.

Gene	Temperature (°C)	Sequence 5’→3’
NFKB1	59	CCTCTGTGTTTGTCCAGCT (19)
		CCGAAAAATTGGGCATGAGC (20)
RELA	58	CACGAGCTTGTAGGAAAGG (19)
		GCGCTGACTGATAGCCTG (18)
iNOS	59.4	ACAGGAGGGGTTAAAGCTGC (20)
		TTGTCTCCAAGGGACCAGG (19)
CYP1A1	61.8	CTCTTAGGTGCTTGAGAGCCC (21)
		CATCAGCA TCTATGTGGCCC (20)
IL-6	61.4	GCCTTCGGTCCAGTTGCCTT (20)
		GCAGAATGAGATGAGTTGTC (20)
IL-1b	57.9	ACAGATGAAGTGCTCCTTCCA (21)
		GTCGGAGATTCGTAGCTGGAT (21)
GPX-1	59.8	TTATGACCGACCCCAAGCTCA (21)
		ATGTCAATGGTCTGGAAGCGG (21)
GPX-2	57.3	GGAGAATGAACCCAAGCGAA (20)
		CAGGTTTGTCACAGCCAGTGAT (22)
GPX-3	59.8	TCTCATCCCATGTCCACCATG (21)
		TGCATCCATTTGTGCCAGG (19)
GPX-4	59.8	AGAGATCAAAGAGTTCGCCGC (21)
		TCTTCATCCACTTCCACAGCG (21)
GPX-5	57.9	TCCTTCCACGACAATGGTTCA (21)
		TGTGACTGTGACCCCATTGCT (21)
GPX-6	59.8	CAGAAACCCCACCTCACATGA (21)
		TGCCATGACCTGAATGCACT (20)
GPX-7	57.9	TTGGTCCCATCATTCTTGTGG (21)
		GGCTGGTGATTCACTGGTCAA (21)
18S	60	GGCTGGTGATTCACTGGTCAA (21)
		GTAGTTTCTCAGGCTCCCTCTC (22)

## Supplementary Materials

Supplementary materials can be accessed at: http://www.mdpi.com/1420-3049/21/3/319/s1.
