# Supplementary material for: Differential Response of Two Human Breast Cancer Cell Lines to the Phenolic Extract from Flaxseed Oil
Source: Molecules. 2016 Mar 8;21(3):319. doi: 10.3390/molecules21030319 (PMC6274312; doi:10.3390/molecules21030319)
Supplement: Supplementary file 1 [file molecules-21-00319-s001.pdf]

Supplementary Materials: Differential Response of Two Human Breast Cancer Cell Lines to the Phenolic Extract from Flaxseed Oil

Angela Sorice, Eliana Guerriero, Maria Grazia Volpe, Francesca Capone, Francesco La Cara, Gennaro Ciliberto, Giovanni Colonna and Susan Costantini

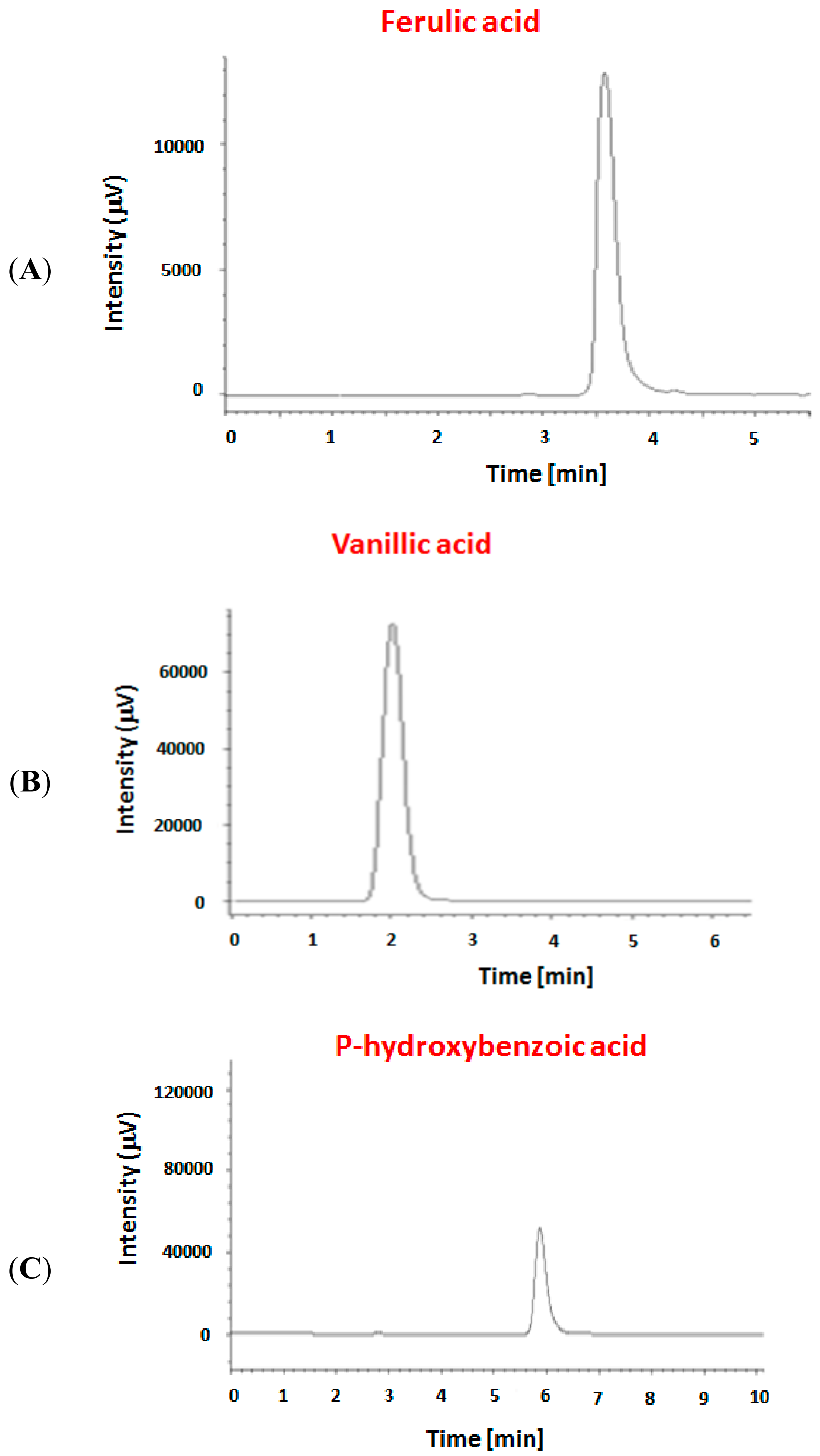

Figure S1. Chromatograms of ferulic acid, vanillic acid and *p*-hydroxybenzoic acid.

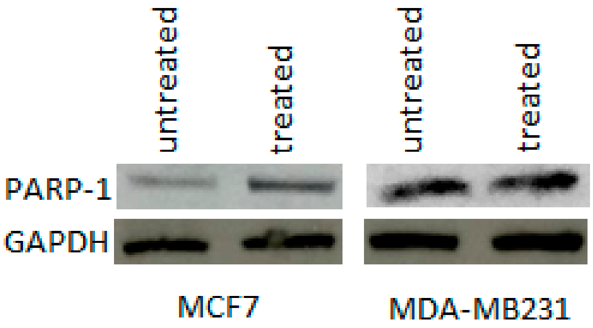

Figure S2. PARP-1 cleaved protein expression analysis by western blotting in MCF7 and MDA-MB231 cell lines before and after treatment with 63 µg/mL and 64.5 µg/mL of phenolic extract from FS oil, respectively. GAPDH was used as loading control.

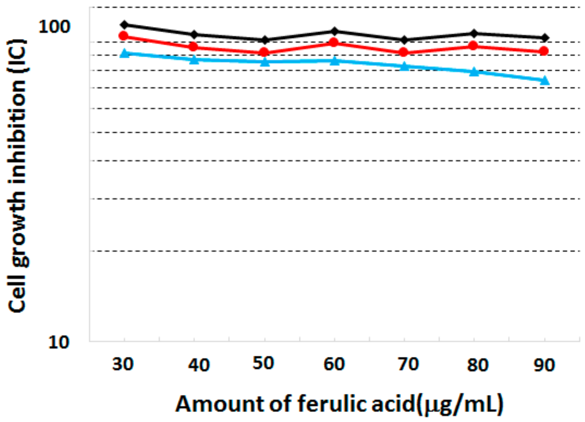

Figure S3. Cytotoxicity assay. We show the cell growth inhibition after 48 h of treatment with different amount of ferulic acid from FS oil on normal human breast epithelial cells, MCF-10A (in black), and two human breast cancer cells, MCF7 (in cyan) and MDA-MB231 (in red).
